# Supplementary material for: Immunogenicity and Safety of Modified Vaccinia Ankara (MVA) Vaccine—A Systematic Review and Meta-Analysis of Randomized Controlled Trials
Source: Vaccines (Basel). 2023 Aug 24;11(9):1410. doi: 10.3390/vaccines11091410 (PMC10536351; doi:10.3390/vaccines11091410)

Supplemental Figure S1: Risk of bias assessment

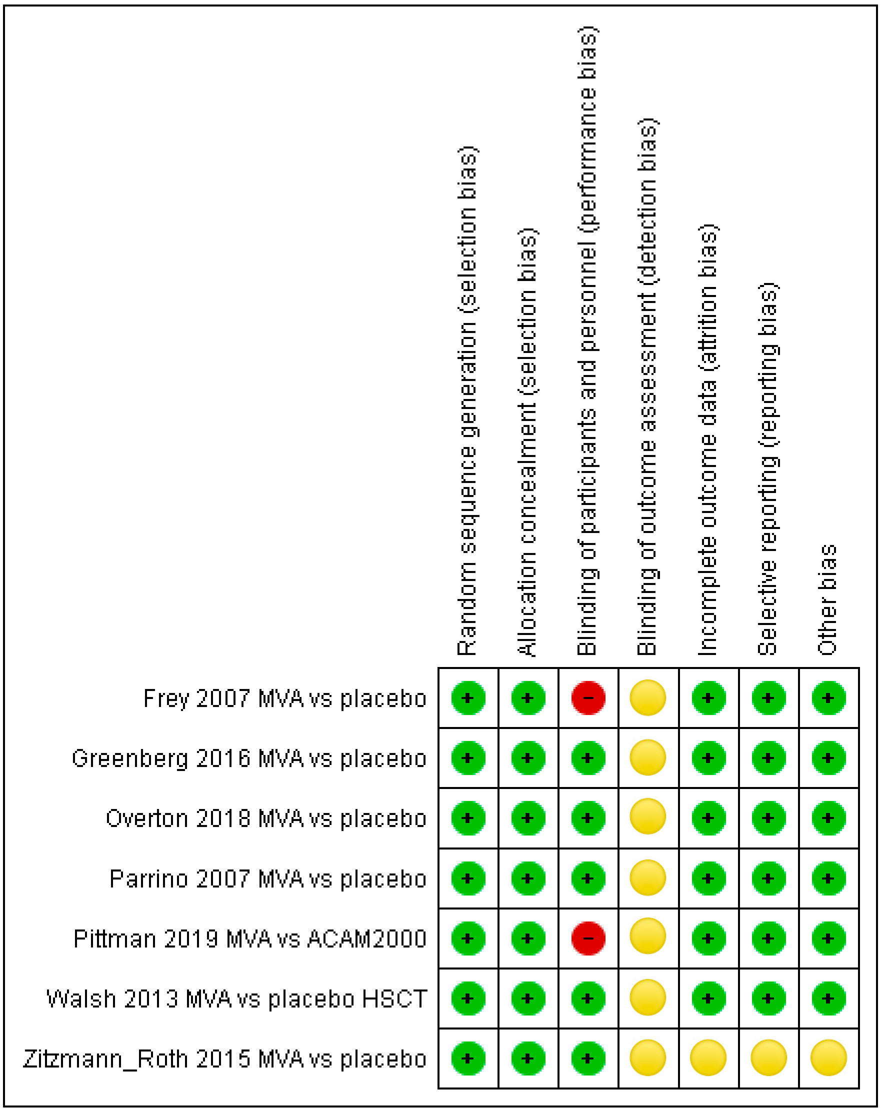

Supplement: Supplementary file 1 [file vaccines-11-01410-s001.zip › Supp_Figure_S1_ROB.pdf]
