# Supplementary material for: Immunogenicity and Safety of Modified Vaccinia Ankara (MVA) Vaccine—A Systematic Review and Meta-Analysis of Randomized Controlled Trials
Source: Vaccines (Basel). 2023 Aug 24;11(9):1410. doi: 10.3390/vaccines11091410 (PMC10536351; doi:10.3390/vaccines11091410)
Supplement: Supplementary file 1 [file vaccines-11-01410-s001.zip › Supp_Table_S1_AESI.pdf]

Supplemental Table S1: Definition of adverse events of special interest (AESI) in the meta-analysis included trials

**Study ID**	**Definition of AESI**
Frey 2007 [21]	No specific definition provided
Greenberg 2016 [15]	Any cardiac symptoms and/or ECG changes determined to be clinically significant or troponin I elevations >X2 upper limit of normal
Overton 2018 [20]	Any cardiac symptoms developed since the first injection, clinically significant ECG changes and troponin I elevations >X2 upper limit of normal
Parrino 2007 naïve [16]	No specific definition provided
Parrino 2007 immune [16]	No specific definition provided
Pittman 2019 [14]	Any cardiac sign or symptom developed since the first injection, any ECG changes determined to be clinically significant or troponin I elevations >X2 upper limit of normal
Walsh 2013 [17]	No specific definition provided
Zitzmann- Roth 2015 (and a following publication – Ilchmann 2023) [19,22]	Any cardiac symptom, clinically significant ECG changes or elevated cardiac enzymes (specifically troponin I elevated above upper limit of normal)
